# Gender, Anxiety, and Legitimation of Violence in Adolescents Facing Simulated Physical Aggression at School

**DOI:** 10.3390/brainsci11040458

**Published:** 2021-04-03

**Authors:** Marina B. Martínez-González, Yamile Turizo-Palencia, Claudia Arenas-Rivera, Mónica Acuña-Rodríguez, Yeferson Gómez-López, Vicente J. Clemente-Suárez

**Affiliations:** 1 Department of Social Science, Universidad de la Costa, Barranquilla 080001, Colombia; yturizo1@cuc.edu.co (Y.T.-P.); carenas@cuc.edu.co (C.A.-R.); macuna6@cuc.edu.co (M.A.-R.); ygomez22@cuc.edu.co (Y.G.-L.); 2 Faculty of Sport Sciences, Universidad Europea de Madrid, 28670 Villaviciosa de Odón, Spain; vctxente@yahoo.es; 3 Grupo de Investigación Cultura, Educación y Sociedad, Universidad de la Costa, Barranquilla 080001, Colombia

**Keywords:** bullying, moral disengagement, violence, disruptive behavior, peer aggression, social rules, socialization, externalizing symptoms

## Abstract

We analyzed gender and anxiety differences in middle school students facing a physical peer aggression situation. The participants were 1147 adolescents aged between 12 and 18 years (male: *n* = 479; female: *n* = 668) who watched a 12 s animation representing the situation and filled out a questionnaire to analyze the legitimation of violent behaviors and anxiety levels. We registered their decisions to solve the situation using a categorical scale that included assertive, avoidant, aggressive, submissive, and supportive behaviors. Gender was not associated with the adolescent’s behaviors in facing a simulated peer aggression situation. However, male teenagers tended to perceive adults as sanctioners and neutrals; those who used the diffusion of responsibility and dehumanization to justify their behavior also showed a higher state of anxiety. Female teenagers who expected legitimation from their peers, presented higher anxiety as well. Educational interventions may use these results, helping adolescents to understand that their acts have substantial implications in the lives of others. It is essential to develop group interventions that modify how adolescents manage their conflicts and change gender stereotypes that significantly impact health. We highlight the need for linking families in educational programs facing the challenges of transforming the legitimization of violence in parental practices.

## 1. Introduction

Legitimation is a psychological construct used to analyze authority, power, blind obedience, sociopolitical violence, individual/state relationship, and social protest [1]. In the context of violence, this concept explains the justifying discourse that keeps people willing to commit punishable actions against others [2]. Internalization and institutionalization processes consolidate these beliefs in daily interpersonal relationships, assuming violence as inevitable and even admissible in a group or society [3].

Previous studies about violence legitimization in childhood highlighted the perception of legitimacy to use violence against provocation, based on the authority, and as a persuasive action when the situation is threatening [4,5,6,7]. These studies also analyzed the role of moral disengagement mechanisms and the expectations of legitimation perceived from peers and adults as behavioral determinants [7,8,9].

Regarding the use of moral disengagement mechanisms, Bandura [4,5] postulated eight cognitive mechanisms to maintain a positive self-concept, reducing guilt in immoral actions: (a) moral justification links a violent act to a heroic purpose; (b) euphemistic language reduces the harmful connotation of the act; (c) advantageous comparison minimizes the immoral act, contrasting it with another crueler act; (d) displacement of responsibility identifies an authority as responsible for the acts; (e) diffusion of responsibility is when the action of the group mitigates the perception of one’s own responsibility; (f) distortion of consequences minimizes the harmful effects of a behavior; (g) attribution of blaming refers the victim as provocative; and (h) dehumanization removes people from their human qualities to facilitate mistreatment against them.

Growing up perceiving situations of violence both in the family and in the community has been associated with children’s legitimation of violence [7,8,10,11]. A context that legitimizes violence reduces prosocial behaviors [12] and reduces the negative affect of the anxiety associated with witnessing these events and recognizing its manifestations [13]. For this reason, the social acceptance of violence exposes children to the risk of reproducing violence in their daily relationships [10,11,14], but also in the society that they will constitute in adulthood [15,16].

However, children and adolescents who live in violent situations are exposed to chronic stress that compromises their health [17,18]. Anxiety response refers to different physical and mental manifestations that are not attributable to real dangers and appears as crises or diffuse states [19]. Some authors distinguish between state anxiety and trait anxiety. The first consists of a transitory state facing current events with a higher probability of change over time. The second is considered more stable and durable [20]. These anxiety states could vary in intensity and durability according to the different situations or evolutionary stages that everyone goes through. However, adolescence is the time in life where there is a greater willingness to generate anxiety, with social, emotional, and behavioral effects [21,22]. These difficulties appear in building conflictive interpersonal relationships, less emotional control, rejection of criticism, little acceptance among peers, and victimization [23,24].

Previous researchers found higher levels of anxiety in women, especially in adolescence and childhood [25]. Likewise, a higher incidence of state anxiety has been reported in women than men, associated with maturational and reproductive processes (premenstrual cycle, pregnancy, menstrual delays, and the social pressure of adolescence, among others), and a higher rate of related negative affect with stress, anxiety, and depression [26,27,28]. Many of these situations involve school conflicts as the main interaction scenario in adolescence, a stage in which gender differences associated with aggression have been reported [29,30]. In this line, male teenagers are more aggressive than female teenagers when facing problems, tending to engage in antisocial behaviors as physical and verbal abuse and rule violations. On the other hand, female teenagers seem to have a prosocial orientation and inclination to solve problems assertively, empathize, and be concerned with others. However, new evidence has found no gender differences related to aggression manifestations [31], which could be associated with a generational and cultural change in parenting and relationship patterns [32].

The present research aimed to analyze gender and anxiety differences in middle school students’ behavior facing a simulated physical peer aggression situation. The study hypotheses were (i) the gender of the participants, offenders, and the authorities would modulate the adolescent’s behaviors in a simulated peer aggression situation; and (ii) the legitimization of violence would be present in males and participants with higher anxiety levels.

## 2. Materials and Methods

A total of 1147 volunteer adolescents participated in the present research, aged between 12 and 18 years (male: *n* = 479; M = 16.32; SD = 1.10; female: *n* = 668; M = 16.27; SD = 0.85), with a stratified random sampling of simple affixation, in which the sample was collected from schools at different socioeconomic levels from the city of Barranquilla (Colombia). The procedure was conducted following the Helsinki Declaration (revised in Brazil, 2013) and approved by the university ethical committee (approval code 094).

The data were collected anonymously. Before participating, all participants, parental or guardian, and their professors were informed about the experimental procedures, indicating the right to withdraw from the study at any time and providing written informed consent.

### 2.1. Procedure

As a laboratory investigation, this study used animations that simulated physical peer aggression at school to assess different reactions from participants. Previous researchers have effectively used simulated scenarios of violence to assess participants’ responses [33,34,35,36,37].

The adolescents were contacted in different schools. The final sample was conformed for those whose parents consented to participate. They completed the evaluation task in a computer room, in groups of 30 people, sitting randomly to face the different situations presented. First, they read the purpose of the study and gave consent to participate. Next, the instructions appeared, and the participants answered demographic questions. Then, instructions to watch the video and answer related questions were given.

The research was carried out with a cross-sectional evaluation using a multifactorial randomized block design. The adolescents were placed according to their gender in four possible stimulus combinations, as detailed below.

After observing the stimulus video, they answered how they would react to that situation and questions related to moral disengagement mechanisms to justify their action. In the end, all of the adolescents answered the anxiety questionnaires.

### 2.2. Instruments

**Figure 1:**
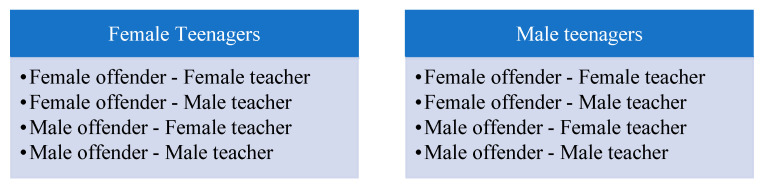
We used a randomized block design in the study, considering the gender of the participants and the animated version of the offenders and teachers.

**Figure 2:**
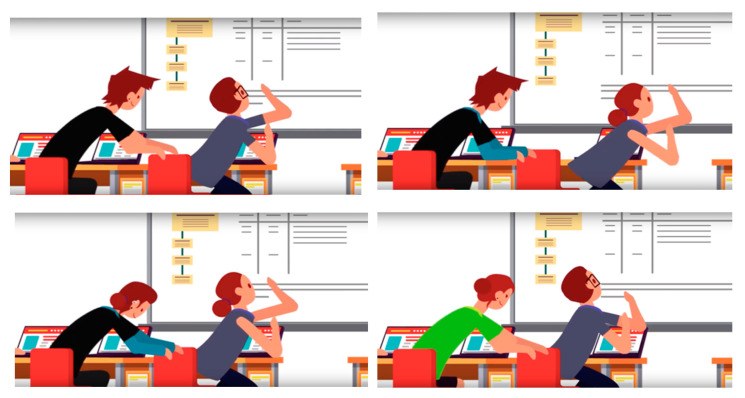
Images of stimulus simulating a peer’s physical aggression at school. In each box, the offender’s character is on the left, and the participant’s character on the right.

An animation with a simulated physical peer aggression situation was shown. Participants watched a 12 s online animation representing a physical violence situation from peers at school. There were four different stimuli, with the gender of the offender and the teacher varying (see Figure 1). The stimulus consisted of an animation with a voiceover describing the situation to generate the participant’s identification with the main character. The scene showed a group of students and the teacher in the classroom; then, the teacher went out to answer a call. In his/her absence, one of the students, described as a bully, pushed the character identified with the participant. Some images from the animation are presented in Figure 2.

After this, questions about the reaction in facing the situation and its justifications were presented. These questions were inspired by moral disengagement mechanism theory [4,5,38], and include questions about the legitimation of violence expected from their peers and adults as mediators in the conflict. The answers were registered using a categorical scale that included assertive, avoidant, aggressive, submissive, and supportive behaviors; then, the answers were integrated to analyze if participants tended to attack or not (assertive, avoidant, submissive, and supportive categories were integrated as “no attack” and the aggressive responses as “attack”).

The State-Trait Anxiety Inventory for children and adolescents was used to measure anxiety [39]. It is composed of two scales, the first to measure state anxiety, containing 20 items, and the second one to measure trait anxiety, with 20 more items. An example of a question is: “I am worried about things at school.”

### 2.3. Statistical Analysis

JASP statistical software was used to analyze the data. The chi-square test was used to analyze the reactions according to gender, and ANOVA was used to analyze differences in anxiety levels according to the participant’s gender and their reactions facing the proposed situation. The level of significance was set at *p* ≤ 0.05.

## 3. Results

### 3.1. Adolescents’ Behaviors in Facing the Simulated Physical Peer Aggression Situation by Gender

We found that 11.5% of males and 12.3% of females decided to attack as a reaction to the stimulus (Table 1). No significant differences were found in the tendency to attack by the participant’s gender (*p* = 0.683), aggressor’s gender (*p* = 0.06), teacher’s gender (*p* = 0.185), or the combination of the aggressor’s and teacher’s gender (*p* = 0.137). There were also no significant differences in state anxiety (*p* = 0.579) and trait anxiety (*p* = 0.72) by gender.

### 3.2. Moral Disengagement Mechanisms Used by Gender

Regarding the mechanisms of moral disengagement used by the participants, no differences by gender were found for moral justification (*p* = 0.336), advantageous comparison (*p* = 0.352), displacement of responsibility (*p* = 0.364), distortion of consequences (*p* = 0.458), attribution of blaming (*p* = 0.88), or dehumanization (*p* = 0.077). Significant differences by gender were found for the mechanisms of euphemistic language and diffusion of responsibility, with males presenting both mechanisms in a higher proportion than females (Table 2).

#### Anxiety Related to Diffusion of Responsibility and Dehumanization by Gender of the Participants

Male participants showed higher state anxiety than female participants when the used diffusion of responsibility and dehumanization mechanisms (Table 3). No significant differences in state anxiety or trait anxiety by gender were found when the participants used euphemistic language (*p =* 0.304), as well as in trait anxiety for the mechanisms of diffusion of responsibility (*p =* 0.718) and dehumanization (*p* = 0.834).

### 3.3. Legitimation of Violence Expected from Peers and Adults by Gender

Significant differences by gender in the legitimization of violence expected from peers and adults were found (Table 4). There was a lack of legitimization of violence expected from peers. However, females perceived them as legitimizers of their violent reaction. The perception of peers as sanctioners was minimal for both males and females. There was a lack of legitimation of violence perceived in adults, especially in females. Males were slightly more likely to perceive adults as sanctioners and neutral than females.

#### Anxiety Related to Legitimation of Violence Expected from Peers and Gender of the Participants

Finally, trait anxiety was significantly higher in females, especially those who identified peers as legitimizers of their reaction (Table 5). No significant differences by gender were found in trait anxiety (0.663) and state anxiety (0.578) when adults were perceived as legitimizers of violence. No significant differences were found in state anxiety (*p* = 0.257) for the legitimation expected from peers associated with the gender of the participants.

## 4. Discussion

This study aimed to analyze gender and anxiety differences in middle school students’ behavior facing a simulated physical peer aggression situation. The hypothesis (i) was not confirmed, since gender was not associated with the adolescent’s behaviors in a simulated peer aggression situation; hypothesis (ii) was confirmed, since males presented higher moral disengagement mechanisms to justified violent reactions and a higher state anxiety when they used diffusion of responsibility and dehumanization mechanisms to justify their behavior.

The absence of gender differences in the use of violence in the present research was in line with previous research in this area [31]. Early studies about the prevalence of antisocial behavior in boys versus girls reported stronger genetic influences in girls and stronger environmental influences in boys. However, later meta-analyses found that antisocial behavior was equally heritable, but its etiology could differ across sex [40].

Generational and cultural changes in parenting and relationship patterns could impact new relationship forms that normalize violence without gender differences [29,32].

In this study, females evidenced a slightly higher expectation of legitimization from peers than males. This result coincides with previous studies, where females tend to be more concerned with social approval, afraid of abandonment [41,42], and worried about evaluation from their peers [28,43]. Our results also evidence that females with higher trait anxiety expected more legitimation from their peers. The higher trait anxiety levels could make them understand the violence as a catharsis, legitimizing it [44]. In this line, previous researchers found that girls were more at risk for internalizing adjustment problems as negative affect with stress, anxiety, and depression [26,27], and find adverse interpersonal events more stressful than males [45]. Relative to adults, girls did not perceive them as legitimizers or sanctioners. These results could be explained by adults’ expectations about girls, who tend to evaluate them as less violent than boys [46].

We found that boys evidenced a higher expectation of neutrality and sanction from adults. These results could contradict previous studies that evidenced that many cultural parenting patterns promote male children’s violence to solve conflicts [14]. However, neutrality expectations coincide with those studies, since many parents leave their children to decide when to use violence [7]. In consonant, it was reported that boys experience more advised violence from family, but even from non-family members, including neighbors and peers [10]. This fact could represent a stressful factor regarding the socially expected behavior of men facing conflicts. The social acceptance of violence exposes children to the risk of reproducing it in their daily relationships [10,11,47], and exposes them to chronic stress [17] and posttraumatic stress disorders in young adulthood [18]. In this study, male teenagers showed higher state anxiety associated with using moral disengagement mechanisms, such as diffusion of responsibility and dehumanization.

The diffusion of responsibility considers the group’s role in the perception of individual responsibility for an act [4,5]. In this case, the increase of anxiety shows the possible social pressure experienced by boys facing interpersonal conflicts. Dehumanization is considered the worst violence justification [48], and its use has implications for the development of empathy by perceiving certain human beings as having fewer human qualities [49]. Usually, perceiving the other’s suffering generates aversive sensations, but dehumanization reduces this empathy. Nevertheless, today, there is some doubt about the concept explaining this moral failure related to care about the other’s suffering as not presupposing a cognitive failure to recognize their humanity. Contrarily, this remains an intensely human undertaking [50]. Thus, the link between dehumanization and state anxiety could be evidence of this cognitive contradiction.

Other moral disengagement mechanisms, such as euphemistic language and diffusion of responsibility, showed variations between males and females. Those mechanisms have been found with a strong presence in adolescents, increasing bullying perpetration. The adolescents who recur in these thoughts to justify their actions describe them as not severe and without significant consequences [49], which maintains these behaviors, preventing them from disappearing. Therefore, modification in the adolescent’s perception in this sense appears to be essential to reduce bullying cases.

### 4.1. Limitations

The participants of this study were from Colombia. This country and its population have experienced more than 60 years of internal armed conflict, with consequently high exposure to violent content through the media and in many aspects of daily life. The generalizability of the results to other populations and contexts will need replication through cross-cultural investigations that favor a greater understanding of the phenomenon of the legitimization of violence in adolescence, and its relationships with anxiety.

### 4.2. Prevention and Policy Implications

The results obtained in this research can be used by educational interventions to improve coexistence and programs to change the justification of violent behaviors, helping adolescents to understand that their acts have substantial implications in the lives of others. Likewise, it is essential to develop group interventions that modify how adolescents’ conflicts are managed, as the same as gender stereotypes that have a significant impact on health. Finally, we highlight the need for linking families in educational programs facing the challenges of transforming the legitimization of violence in parental practices.

## 5. Conclusions

The gender of the participants, offenders, and authorities was not associated with the adolescents’ behaviors in a simulated peer aggression situation. Nevertheless, moral disengagement mechanisms, such as euphemistic language and diffusion of responsibility, were higher in males. Male teenagers showed a greater tendency to perceive adults as sanctioners and neutrals; males had higher state anxiety when they used diffusion of responsibility and dehumanization mechanisms to justify their behavior. Female teenagers presented higher trait anxiety when they expected legitimation from peers.

## Figures and Tables

**Table 1:** Comparison by gender for reactions facing the simulated physical peer aggression situation.

		Reaction to Stimulus		Total
		Attack	Does not Attack	
Participant gender				
Male	Count	55.00	424.0	479.0
	% within column	40.1 %	42.0 %	41.8 %
Female	Count	82.00	586.0	668.0
	% within column	59.9 %	58.0 %	58.2 %
Total	Count	137.00	1010.0	1147.0
	% within column	100.0 %	100.0 %	100.0 %
	Chi-Squared Tests	Χ^2^ = 0.167	*p* = 0.683	
Offender gender				
Male	Count	81.00	511.0	592.0
	% within column	59.1 %	50.6 %	51.6 %
Female	Count	56.00	499.0	555.0
	% within column	40.9 %	49.4 %	48.4 %
Total	Count	137.00	1010.0	1147.0
	% within column	100.0 %	100.0 %	100.0 %
	Chi-Squared Tests	Χ^2^ = 3.515	*p* = 0.061	
Teacher gender				
Men	Count	80.00	529.0	609.0
	% within column	58.4 %	52.4 %	53.1 %
Women	Count	57.00	481.0	538.0
	% within column	41.6 %	47.6 %	46.9 %
Total	Count	137.00	1010.0	1147.0
	% within column	100.0 %	100.0 %	100.0 %
	Chi-Squared Tests	Χ^2^ = 1.754	*p* = 0.185	
Offender and Teacher gender combined				
Men-Men	Count	46.00	280.0	326.0
	% within column	33.6 %	27.7 %	28.4 %
Men-Women	Count	46.00	325.0	371.0
	% within column	33.6 %	32.2 %	32.3 %
Women-Men	Count	23.00	155.0	178.0
	% within column	16.8 %	15.3 %	15.5 %
Women-Women	Count	22.00	250.0	272.0
	% within column	16.1 %	24.8 %	23.7 %
Total	Count	137.00	1010.0	1147.0
	% within column	100.0 %	100.0 %	100.0 %
	Chi-Squared Tests	Χ^2^ = 5.534	*p* = 0.137	

Designed by the authors.

**Table 2:** Comparison by gender for moral disengagement mechanisms (euphemistic language, diffusion of responsibility).

Moral Disengagement Mechanisms		Euphemistic Language			Diffusion of Responsibility		
Gender		Undecided	Absence	Presence	Undecided	Absence	Presence
Male	Count	325.0	66.00	88.00	136.0	304.0	39.00
	% within row	67.8 %	13.8 %	18.4 %	28.4 %	63.5 %	8.1 %
	% within column	40.3 %	39.3 %	50.9 %	39.0 %	41.8 %	54.9 %
Female	Count	481.0	102.00	85.00	213.0	423.0	32.00
	% within row	72.0 %	15.3 %	12.7 %	31.9 %	63.3 %	4.8 %
	% within column	59.7 %	60.7 %	49.1 %	61.0 %	58.2 %	45.1 %
Total	Count	806.0	168.00	173.00	349.0	727.0	71.00
	% within row	70.3 %	14.6 %	15.1 %	30.4 %	63.4 %	6.2 %
	% within column	100.0 %	100.0 %	100.0 %	100.0 %	100.0 %	100.0 %
Chi-Squared Tests		Χ^2^	7.007	*p* = 0.030	Χ^2^	6.182	*p* = 0.045

Designed by the authors.

**Table 3:** ANOVA for STAI-E related to diffusion of responsibility and dehumanization by the gender of the participants.

Moral Disengagement Mechanism	Gender	Mean	SD	*n*	F	*p*
Diffusion of responsibility						
Undecided	Male	28.71	2.740	136	5.151	0.006
	Female	28.87	2.770	213		
Absence	Male	28.79	2.743	304		
	Female	28.44	2.598	423		
Presence	Male	30.38	2.889	39		
	Female	28.28	2.517	32		
Dehumanization						
Undecided	Male	29.28	3.351	46	4.003	0.019
	Female	28.12	2.590	41		
Absence	Male	28.77	2.625	405		
	Female	28.62	2.687	592		
Presence	Male	30.11	3.665	28		
	Female	28.26	2.105	35		

Designed by the authors.

**Table 4:** Comparison by gender for the legitimation of violence perceived in peers and legitimation of violence perceived in adults.

		Legitimation from Peers				Legitimation from Adults			
Gender		Neutral	Absence	Presence	Sanction	Neutral	Absence	Presence	Sanction
Male	Count	191.0	241.0	45.00	2.00	29.00	357.0	5.00	88.00
	% within row	39.9 %	50.3 %	9.4 %	0.4 %	6.1 %	74.5 %	1.0 %	18.4 %
	% within column	48.6 %	38.4 %	36.6 %	50.0 %	55.8 %	39.1 %	41.7 %	52.1 %
Female	Count	202.0	386.0	78.00	2.00	23.00	557.0	7.00	81.00
	% within row	30.2 %	57.8 %	11.7 %	0.3 %	3.4 %	83.4 %	1.0 %	12.1 %
	% within column	51.4 %	61.6 %	63.4 %	50.0 %	44.2 %	60.9 %	58.3 %	47.9 %
Total	Count	393.0	627.0	123.00	4.00	52.00	914.0	12.00	169.00
	% within row	34.3 %	54.7 %	10.7 %	0.3 %	4.5 %	79.7 %	1.0 %	14.7 %
	% within column	100.0 %	100.0 %	100.0 %	100.0 %	100.0 %	100.0 %	100.0 %	100.0 %
Chi-Squared Tests		Χ^2^	11.87	*p* =	0.008	Χ^2^	14.33	*p* =	0.002

Designed by the authors.

**Table 5:** ANOVA for STAI-R related to the legitimation of violence perceived in peers and gender of the participants.

Peer Legitimation	Gender of the Participants	Mean	SD	*n*	F	*p*
Neutral	Male	24.69	4.344	191	2.962	0.031
	Female	26.27	4.585	202		
Absence	Male	25.15	4.431	243		
	Female	25.03	4.251	388		
Presence	Male	26.38	4.868	45		
	Female	26.67	4.755	78		

Designed by the authors.

## Data Availability

Data supporting reported results can be found asking directly of the first author.
